# Infrared Imaging Combined with Machine Learning for Detection of the (Pre)Invasive Pancreatic Neoplasia

**DOI:** 10.1021/acsptsci.4c00689

**Published:** 2025-03-20

**Authors:** Danuta Liberda-Matyja, Kinga B. Stopa, Daria Krzysztofik, Pawel E. Ferdek, Monika A. Jakubowska, Tomasz P. Wrobel

**Affiliations:** † Doctoral School of Exact and Natural Sciences, 37799 Jagiellonian University, ul. Łojasiewicza 11, 30-348 Krakow, Poland; ‡ Solaris National Synchrotron Radiation Centre, 37799 Jagiellonian University, ul. Czerwone Maki 98, 30-392 Krakow, Poland; § Malopolska Centre of Biotechnology, 37799 Jagiellonian University, ul. Gronostajowa 7A, 30-387 Krakow, Poland; ∥ Department of Cell Biology, Faculty of Biochemistry, Biophysics and Biotechnology, 37799 Jagiellonian University, ul. Gronostajowa 7, 30-387 Krakow, Poland

**Keywords:** digital pathology, histopathology, infrared imaging, mouse models of pancreatic cancer, machine learning, pancreatic intraepithelial neoplasia, pancreatic cancer

## Abstract

With the challenge of limited early stage detection and a resulting five-year survival rate of only 13%, pancreatic ductal adenocarcinoma (PDAC) remains one of the most lethal cancers. Replacing the high-cost and time-consuming grading of pancreatic samples by pathologists with automated diagnostic approaches can revolutionize PDAC detection and thus accelerate patient admission into the clinical setting for treatment. To address this unmet diagnostic need and facilitate the shift of tissue screening toward automated systems, we combined stain-free histology—specifically, Fourier-transform infrared (FT-IR) imaging—with machine learning. The obtained stain-free model was trained to distinguish between normal, benign, and malignant areas in analyzed specimens using hematoxylin and eosin stained pancreatic tissues isolated from KC (Kras^G12D/+^; Pdx1-Cre) or KPC mice (Kras^G12D/+^; Trp53^R172H/+^; Pdx1-Cre). Due to the pancreas-specific mosaic expression of the mutant *Kras* and *Trp53* genes, changes in pancreatic tissues of this mouse model of PDAC closely mirror the gradual transformation of normal pancreatic epithelia into (pre)­malignant structures. Thus, this mouse model provides a reliable representation of human disease progression, which we tracked in our study with a Random Forest classifier to achieve accurate detection at the cellular level. This approach yielded a comprehensive model that distinguishes normal pancreatic tissues from pathological features such as pancreatic intraepithelial neoplasia (PanIN), cancerous regions, hemorrhages, and collagen fibers, as well as a streamlined model designed to rapidly identify normal tissues versus pathologically altered regions, including PanINs. These models offer highly accurate diagnostic tools for the early detection of pancreatic malignancies, thus significantly improving the chance for timely therapeutic intervention against PDAC.

## Graphical abstract

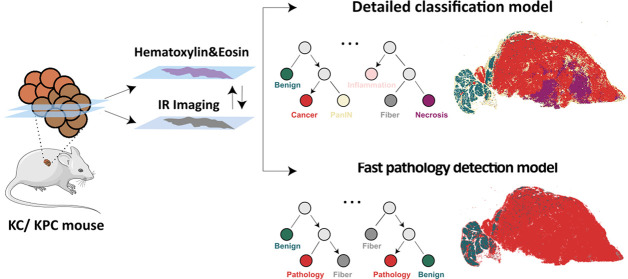

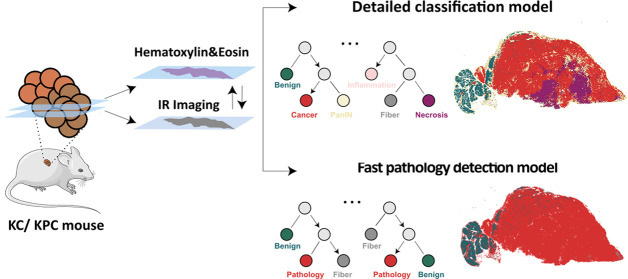

Pancreatic ductal adenocarcinoma (PDAC) is the most prevalent form of pancreatic cancer, accounting for over 90% of all diagnosed cases. Despite clinical advancements, PDAC remains a deadly disease, with a five-year survival rate of only 13%.^1^ Alarmingly, it is projected to become the second most fatal cancer in the US by 2030.^2,3^ Detecting pancreatic cancer in its early stages is vital for predicting patient outcomes. According to information from the US cancer surveillance database covering the years 2004–2016, up to 83.7% of those diagnosed with PDAC in the very early stage 1A survive for five years. However, early stage detection is rare, and in stark contrast, the survival rate diminishes to approximately 10% for individuals diagnosed with advanced-stage PDAC.^4^

In both clinical settings and preclinical studies aimed at testing new therapeutic strategies, the histopathological assessment that differentiates between healthy tissue, premalignant dysplasia, and advanced PDAC – still largely depends on human evaluation. However, the sheer volume of this task, combined with the need to process a large number of samples, highlights the growing need for more automated approaches. These advancements will not only enhance diagnostic precision but also speed up treatment initiation, ultimately improving patient outcomes by ensuring timely and accurate therapeutic interventions.

In recent years, infrared (IR) imaging combined with machine learning has been extensively developed as a method for cancer detection and diagnosis in tissue samples.^5–9^ Fourier-transform infrared (FT-IR) imaging derives information about the sample’s chemical composition in the form of spectra and images while being nondestructive for the analyzed sample. Based on collected spectra of tissue, components (biomolecules) such as proteins, lipids, nucleic acids, and carbohydrates can be recognized.^10^ Machine learning algorithms serve as a tool for the recognition of pathological changes in tissues based on collected IR data. First, the model is trained on a known and very well-defined set of data assigned to different tissue classes (e.g., benign, and cancerous tissue).^11^ Once the model is properly fitted to the analyzed data by tuning its hyperparameters, predictions on test tissues can be done. Random Forest is a robust machine learning algorithm that is relatively resistant to overfitting. It operates by constructing multiple decision trees during training and combining their outputs to improve predictive accuracy. Additionally, the model can identify which features are the most important in the prediction process. These highly important features can then be utilized to develop a rapid cancer detection model, as demonstrated in our previous research.^7,12^ For rapid model creation, selected frequencies can be measured using a system equipped with Quantum Cascade Lasers (QCLs). QCLs allow for the measurement of discrete frequencies, significantly reducing measurement time and improving the feasibility of adapting conventional FT-IR technology for clinical applications.^13,14^

In our previous study, devoted to human pancreatic tissue classification, we successfully developed an IR imaging-based histopathological machine learning model using 600 needle biopsies, achieving excellent performance (with Area Under the Receiver Operating Curve, AUC, values close to 1) on the sample and pixel levels.^15^ We also created a detailed model for detecting and grading premalignant dysplasia in human tissues.^16^ Our research primarily focused on low-grade dysplasia, demonstrating the high potential of this method for detecting and grading premalignant changes in the pancreas. However, to fully understand the progression of precancerous and cancerous conditions, it is essential to utilize mouse-based models that account for specific driver mutations.

The current understanding of PDAC development suggests that it originates from noninvasive precursor lesions arising in the exocrine component of the pancreas, specifically from pancreatic duct cells and acinar cells.^17,18^ The most common precursor lesion, accounting for up to 90% of PDAC cases is pancreatic intraepithelial neoplasia, PanIN.^19,20^ PanINs develop due to the accumulation of driver mutations within the exocrine pancreatic epithelia. The most commonly mutated gene is the v-*K*_i_-ras2 Kirsten rat sarcoma viral oncogene (*KRAS*), which is altered in over 90% of PDAC cases.^21,22^ *KRAS* mutation alone is usually insufficient to trigger progression to invasive disease; therefore additional mutations in genes such as in tumor protein P53 (*TP53*), cyclin-dependent kinase inhibitor 2A (*CDKN2A*), or SMAD protein family member 4 (*SMAD4*) are typically required for the disease to advance^19,22–24^ (Figure 1).

**Figure 1:**
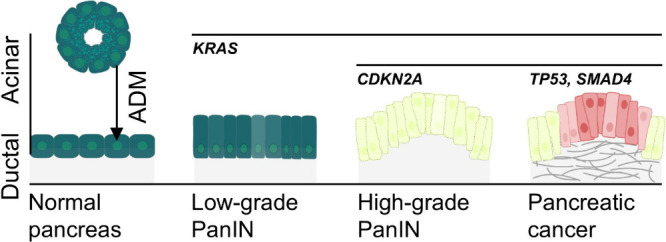
Initiation and progression of PDAC. The *KRAS* mutation triggers acinar-to-ductal metaplasia (ADM), an early event in pancreatic tumorigenesis. Subsequently, pancreatic intraepithelial neoplasia (PanIN) develops. With the accumulation of additional mutations in tumor suppressor genes such as *CDKN2A*, *TP53*, and *SMAD4*, PanIN lesions can progress to high-grade PanINs and eventually invasive PDAC.

In this study, we employed the well-known mouse model of PDAC, KPC mice (Kras^G12D/+^; Trp53^R172H/+^; Pdx1-Cre), which accurately mirrors the initiation and progression of the human disease.^19^ In this model, the Pdx1-guided Cre recombinase activates the expression of mutant *Kras* (Kras^G12D/+^) and *Trp53* (Trp53^R172H/+^) specifically in the pancreas, leading to neoplasm formation and subsequent progression to PDAC within 2–10 months of the mouse lifespan. Additionally, we used mice bearing only the *Kras* mutation (Kras^G12D/+^; Pdx1-Cre), which develop various stages of PanINs without progressing to PDAC.

Using infrared imaging combined with machine learning (Figure 2), we have developed a detailed classification model capable of recognizing normal pancreatic tissue, PanINs, cancer, blood, necrosis, inflammation, and collagen fibers (Figures 3 and 4). We also proposed a model for rapid pathology detection, featuring a reduced number of spectral variables, designed to differentiate between normal pancreatic tissue and pathologically altered tissue (that is PanINs alone or PanINs with invasive cancer), as well as fibrotic regions (Figures 5 and 6).

**Figure 2:**
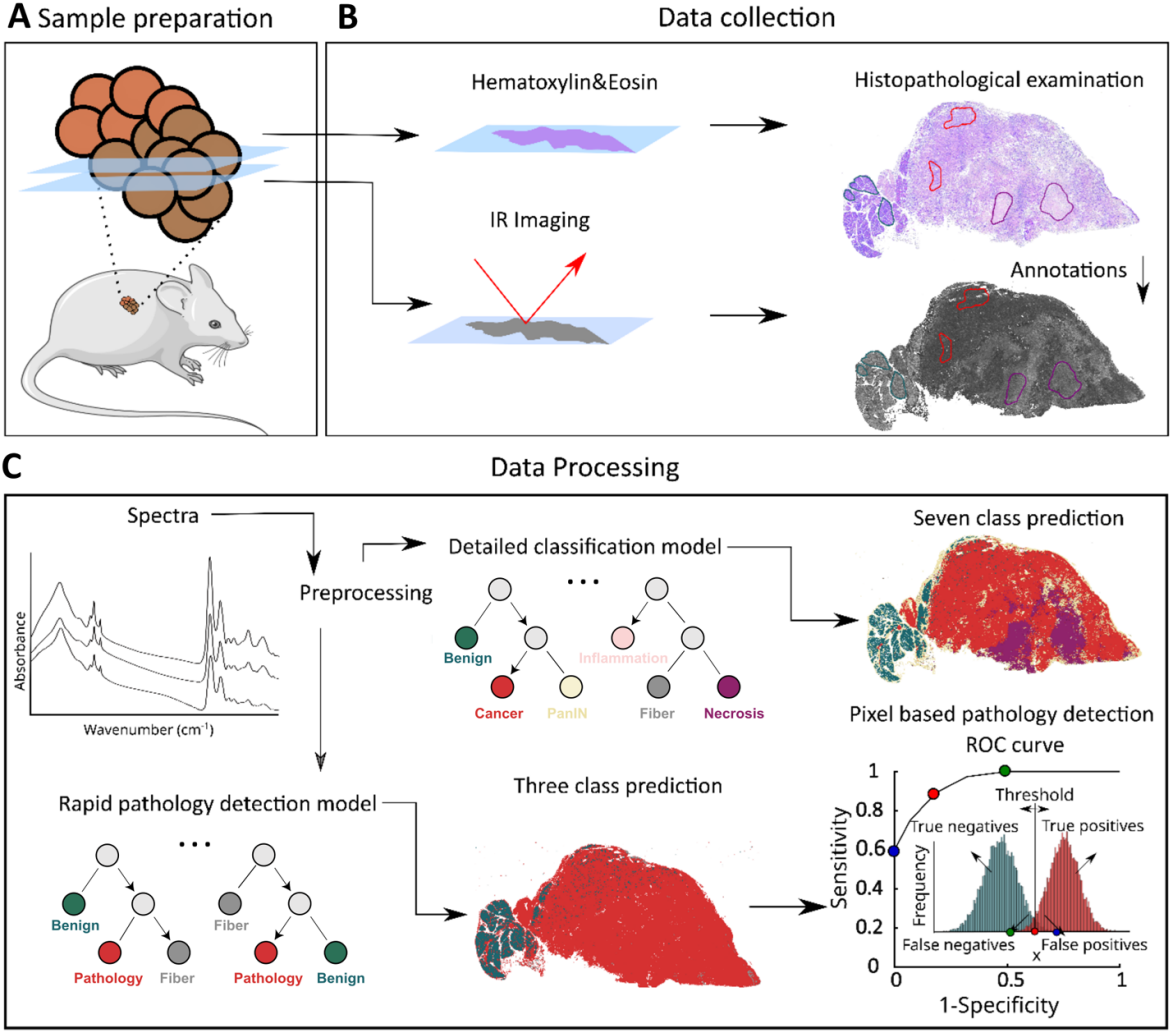
Graphical representation of the experimental pipeline: (A) mouse tissue isolation and sample preparation, (B) data collection, and (C) data processing and final predictions.

**Figure 3:**
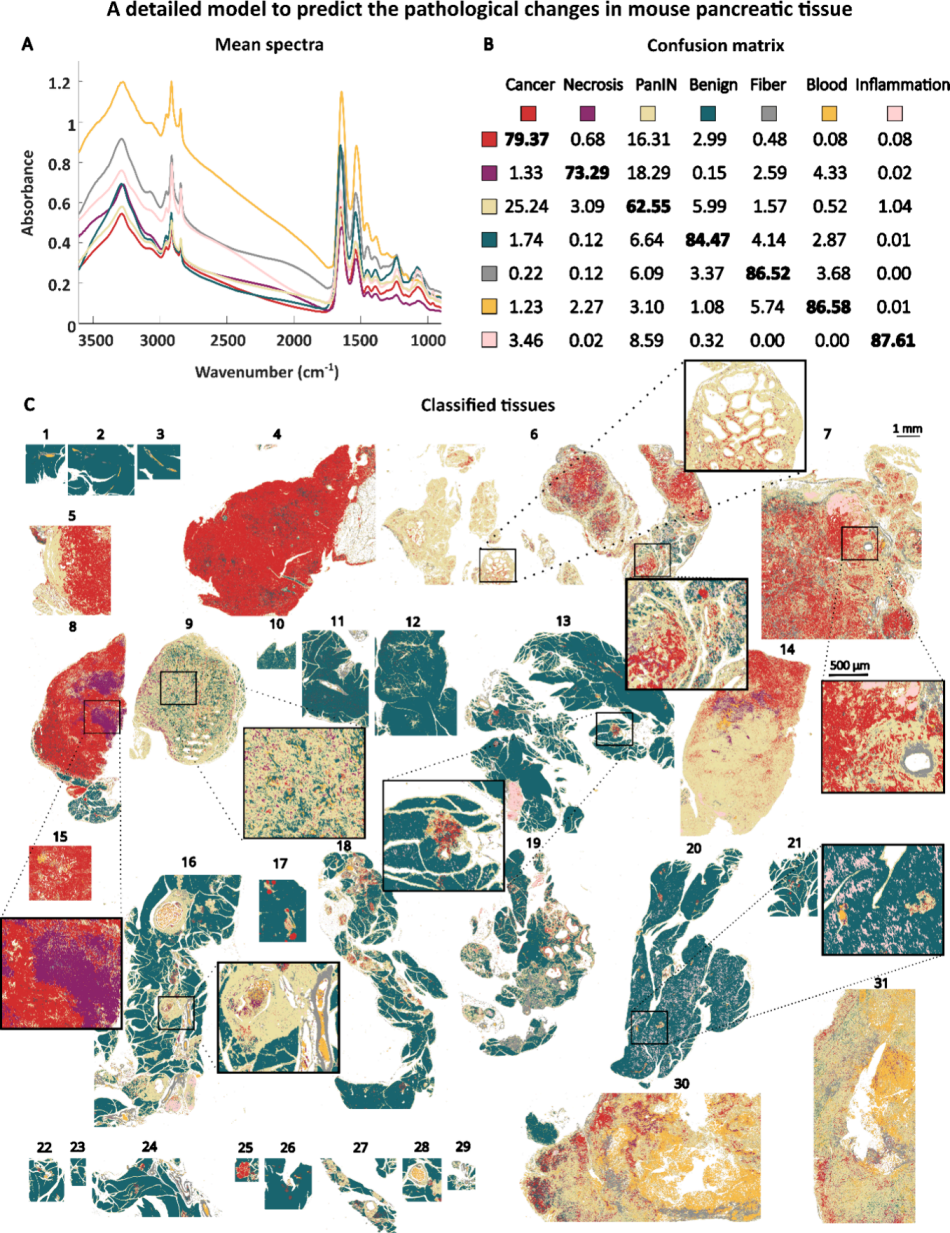
In this comprehensive model, each pixel is assigned to one of seven distinct pathological classes in mouse pancreatic tissue. (A) Mean IR spectra of different classes in mouse pancreatic tissue. Whole tissue images not covered by the zoomed-in elements are presented in Figure S5. (B) Confusion matrix. (C) Representative images showing color-coded class assignments.

**Figure 4:**
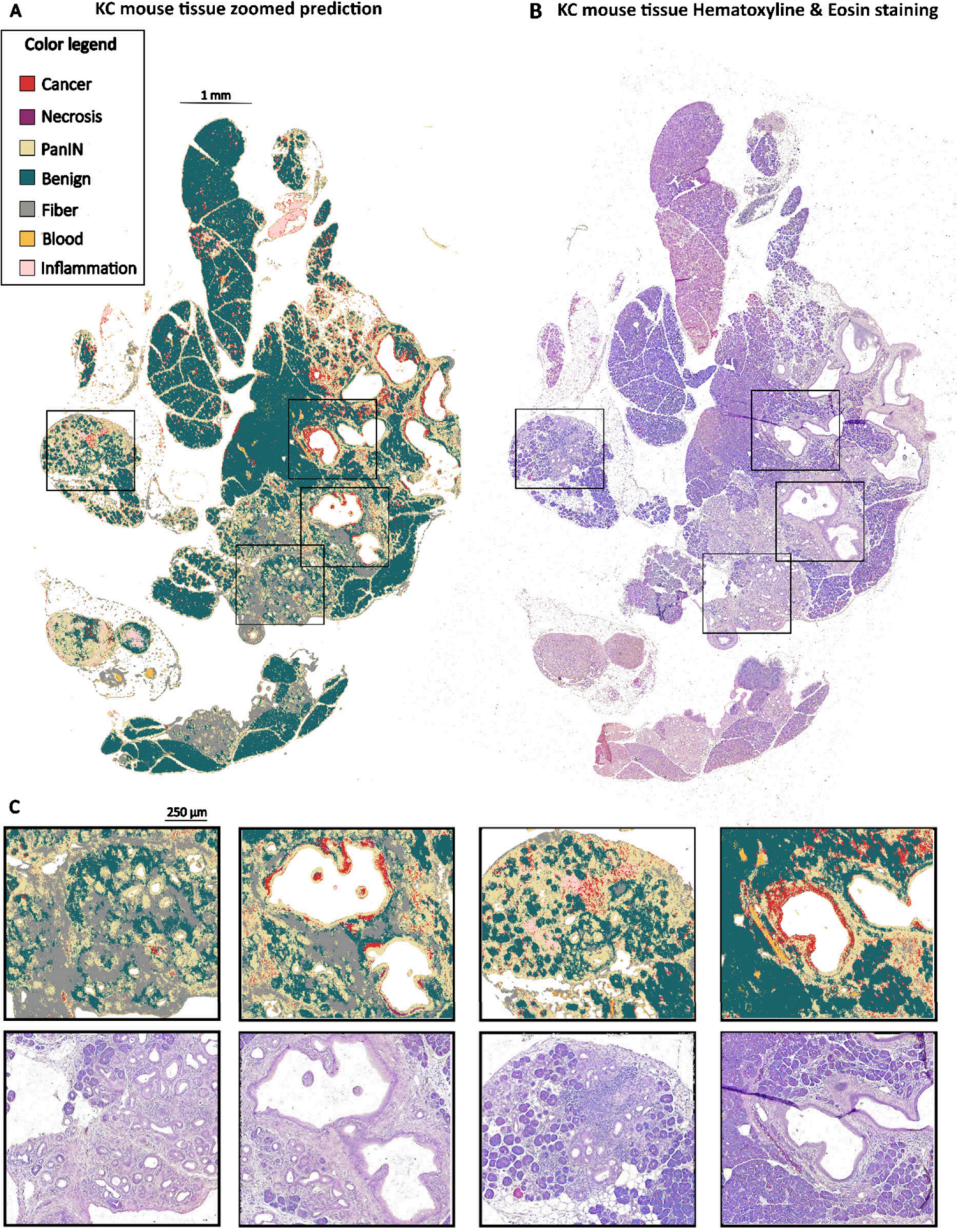
Application of the model in selected mouse tissue (previously presented in Figure 3, tissue no. 19). (A) Color-coded IR image predicting pathological regions in the KC mouse pancreatic tissue, (B) corresponding H&E image, and (C) magnified regions (shown as black frames in A and B).

**Figure 5:**
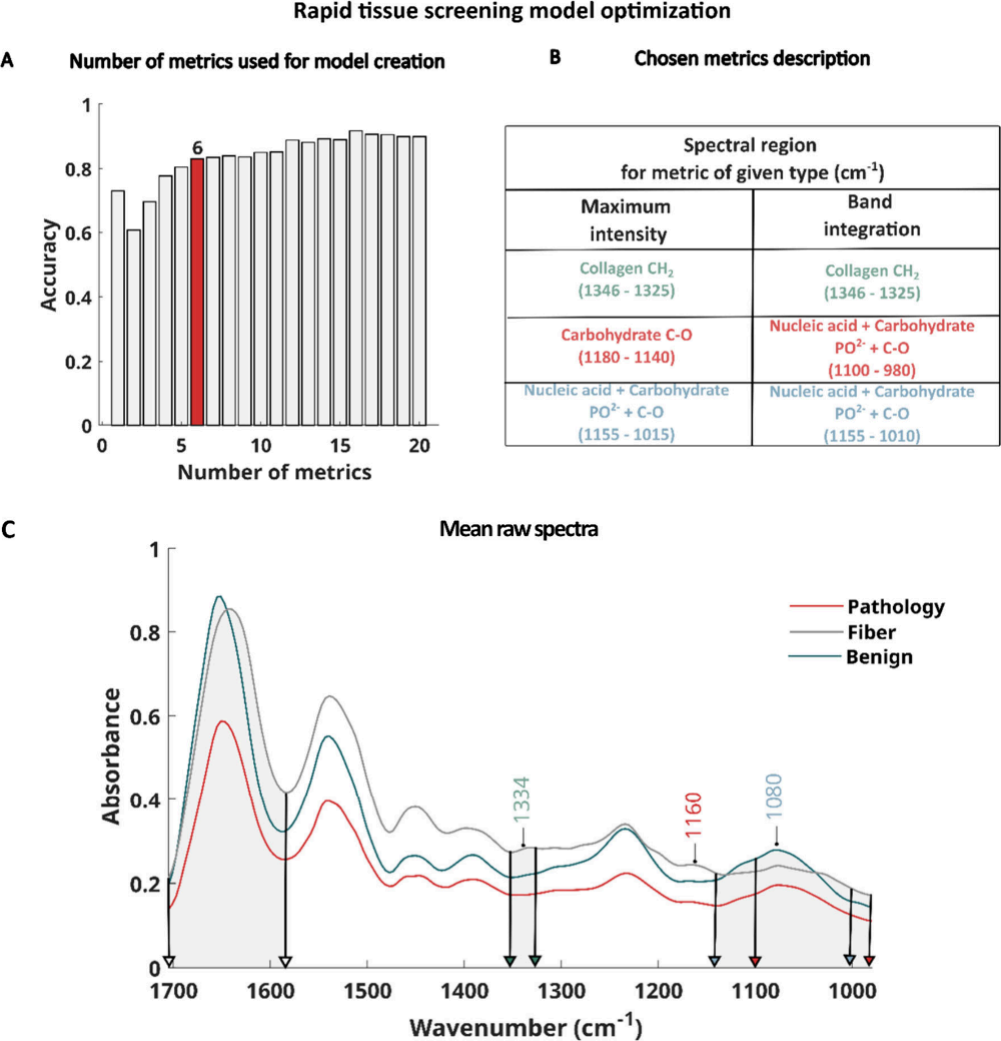
Three-class model for rapid tissue screening to detect pathology (red), fiber (gray), and benign (green) in pancreatic tissue samples: (A) selected number of metrics based on prediction accuracy, (B) biochemical characteristics of the spectra, and (C) mean raw spectra of the analyzed classes (pathology, fiber, benign) with spectral regions corresponding to the selected metrics (areas marked in gray).

**Figure 6:**
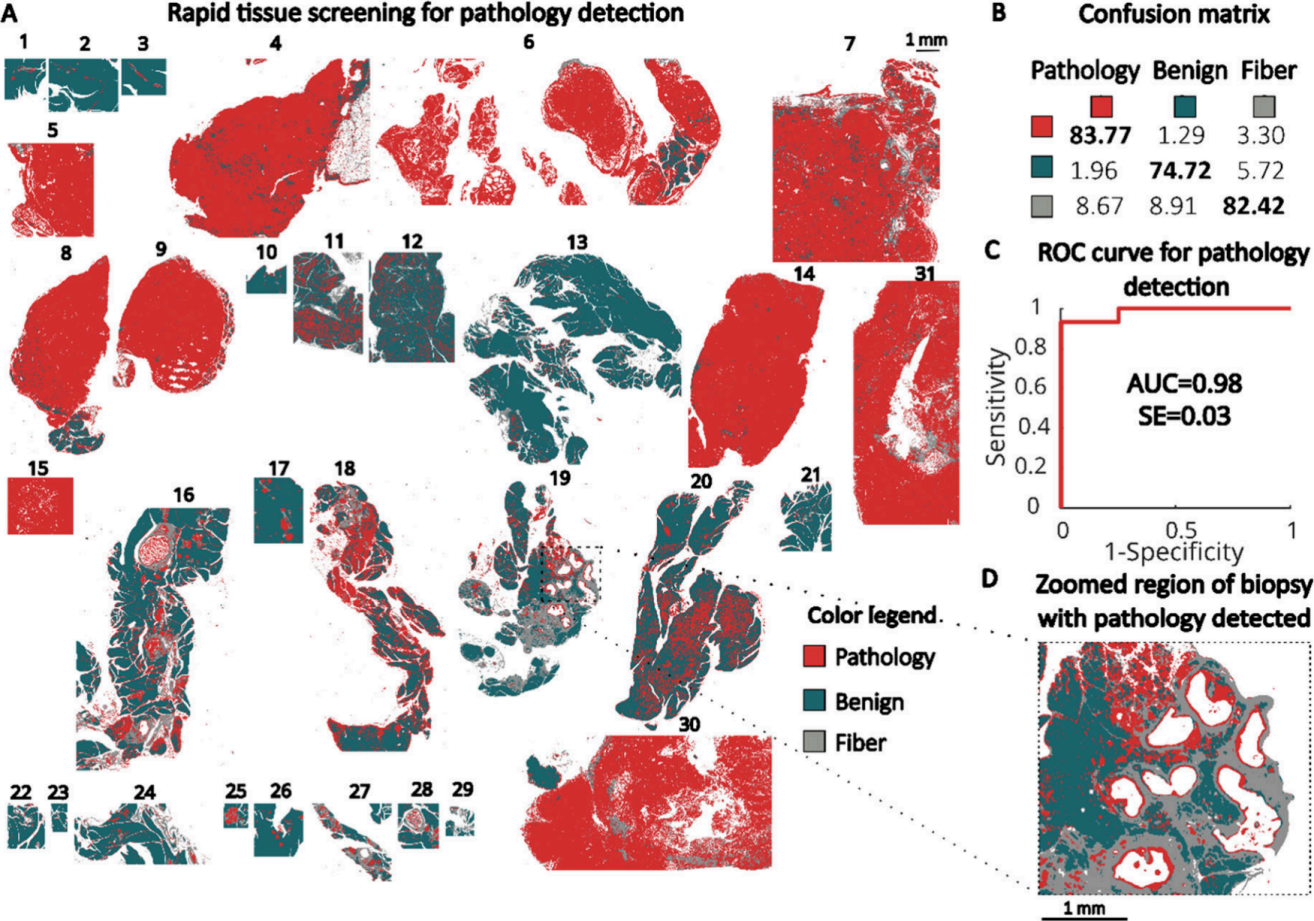
Rapid tissue screening for pathology detection: (A) color-coded IR image predicting pathological regions in mouse pancreatic tissues, (B) pixel-level confusion matrix, (C) receiver operating characteristic (ROC) curve illustrating the performance of classification model, and (D) zoomed region of KC mouse tissue (no. 19) with detected pathology.

## Results and Discussion

Pancreatic tissue sections from KC or KPC mice, 2–10 months old (Figure 2A) were stained with hematoxylin and eosin (H&E) and subjected to histopathological examination (Figure 2B). Adjacent sections were imaged using IR in transflection mode (Figure 2B). Histopathological annotations made on the full H&E slides image were manually transferred to the IR image to define regions of interest (ROIs, Figure 2B). The pixels–representing spectra–within the ROIs were then preprocessed (spectral metrics creation step^25^) and used for Random Forest classification (Figure 2C).^26^ As mentioned in the introduction, the initial detailed tissue classification model was developed based on seven tissue classes: benign (normal/non-neoplastic epithelial pancreatic tissues), cancer (PDAC cells, which may exhibit both epithelial and mesenchymal characteristics), PanIN (precursor lesions to invasive pancreatic cancer), inflammation, fiber, blood, and necrosis (Figure 2C). This classification was done to evaluate the performance of the complex model at the pixel level, as assessed by a confusion matrix. We also evaluated the potential of our model for future translation to a fast quantum cascade laser (QCL) system (Figure 2C).^7,27^ To this end, we created a simplified three-class model for rapid cancer detection. The model’s prediction ability was evaluated using a ROC curve, calculated based on different thresholds (Figure 2C). In this study, the decision criterion *x*, which determines the optimal threshold for classifying mouse tissue as normal or cancerous, was based on the number of pathology-class pixels identified for each mouse (represented by the red histogram in Figure 2C). The area under the ROC curve (AUC) metric was used to assess the model’s diagnostic accuracy, with an AUC of 1 indicating the perfect classification of all tissues.^11^

### Detailed Classification Model

In Figure 3, we presented the results from a detailed, seven-class model classification, detecting pathological changes in pancreatic tissues from KC and KPC mice. Figure 3A displays the raw mean spectra calculated for the analyzed tissue classes, including cancer (red), necrosis (violet), PanIN (yellow), benign (green), fiber (gray), blood (orange), and inflammation (pink). Differences between the preprocessed mean spectra for the benign, cancerous, and PanIN tissue classes are provided in Figure S2. The most prominent differences in absorbance values can be observed in protein regions, Amide A+B 3600–3000 cm^–1^, Amide I 1762–1585 cm^–1^, and Amide II 1585–1473 cm^–1^ (Figure S2D).

The confusion matrix allows us to identify which classes the model finds the most challenging to differentiate, indicating that their chemical compositions are relatively similar. In the confusion matrix shown in Figure 3B, the cancer class was most frequently misclassified as PanIN, and vice versa. This is not particularly surprising given the high biochemical similarities between these two classes, as PanIN lesions are direct precursors to cancer. Mutated PanIN cells subsequently acquire additional mutations and gradually transform into cancer cells, which is reflected by the stochastic classification of some pixels as cancer (Figure 3C, tissue no. 6, left zoom). As PDAC progresses, the cancer cell compartment becomes increasingly prominent (Figure 3C, tissue no. 4, 5, 6 (right zoom), 7, 8, 14, 15).

Importantly, the number of pixels classified as cancer in tissue predominantly composed of PanINs could provide additional information for PanIN grading. For example, in the classification of tissue no. 6 (Figure 3C, left zoom) we observed pathological changes, the majority of which were classified as PanIN, with only a few pixels identified as cancer. Indeed, the histopathological annotations confirmed that this area was a low-grade PanIN, characterized by columnar morphology, mucin-filled cytoplasm, and loss of polarity.^19^ The right zoom of tissue no. 6 shows ducts primarily predicted as cancerous, surrounded by PanINs. This area was annotated as high-grade PanIN/carcinoma in situ, characterized by complete loss of cell polarity and severe nuclear atypia.^19^ Current medical guidelines suggest monitoring low-grade PanINs while recommending surgical removal for high-grade lesions. Therefore, rapid histological identification of high-grade PanINs could aid preventive removal of the lesion before the development of cancer.^28,29^

In general, PanINs were well classified, as demonstrated in predictions for tissues no. 6, 16, 19, 27, and 29. However, as already shown in the confusion matrix, the cancer and PanIN classes were often misclassified. For example, tissues 9, 14, 30, and 31, while histologically classified as cancerous, were predicted as PanINs by the machine learning model. Since our model was trained on only 31 sections, it is possible that the data set contained an insufficient representation of tumor samples, particularly with regard to different tumor types, including a limited number of well- and poorly differentiated tumors. Incorporating a larger number of samples into the model would enhance its robustness to sources of variance unrelated to tissue chemical changes, thereby improving its predictive power and accuracy. A larger data set would strengthen the model’s ability to classify Langerhans islets, differentiate between low- and high-grade PanINs, or distinguish different tumor types. However, creating such a robust model would significantly extend the overall analysis time due to the increased measurement duration and calculational complexity. Additionally, it is important to note that the histopathological analysis and IR measurement were performed on two adjacent tissue slices, not the same one, which may have resulted in slight histological differences, hence the misclassification. Nonetheless, in the remaining tissues, the cancer class was classified correctly (PDAC tissue no. 4–8, and 15).

The healthy exocrine pancreas was correctly predicted in tissues from (i) control animals without *Kras* ^*G12D*^ expression (Kras^+/+^; Pdx1-Cre), tissue no. 1–3, 17; (ii) KC or KPC animals that had not developed significant pathological changes, tissues no. 10–12, 20–24, 26, and (iii) KC or KPC mice in areas surrounding low-grade PanINs, tissues no. 13, 16, 18–20.

Importantly, the seven-class model encompassed not only the presence of cancer cells but also other key features of PDAC such as necrosis, inflammation (indicated by immune cell infiltration), and the extent of fibrosis, all of which are pivotal factors in clinical assessment.

Necrosis, typically observed in poorly vascularized pancreatic tumors, was detected in tissues no. 8 and 14. Collagen fibers were accurately classified both in the pancreatic tumor microenvironment (tissue no. 7, 19, 30, 31) and in blood vessels (zoom on tissue no. 7). Immune cell infiltration, for example in tissue no. 7, was classified with the highest accuracy (87.61% in the confusion matrix, Figure 3B). Focal infiltration - prominent regions of tissue identified as infiltrated by the immune cells, were detected in tissues no. 13, 16, and 19. Furthermore, we also detected other histological features of pancreatic tumors based on our previous classifications, such as hemorrhagic necrosis. Hemorrhagic necrosis, present in tissues no. 30 and 31, was characterized by the presence of blood within PanIN/cancer tissues. All the above classifications were consistent with histological annotations from H&E staining.

Importantly, we were unable to classify Langerhans islets as a separate class in our model. Langerhans islets were instead classified as cancer (for example, red areas present between exocrine lobules in tissue no. 17). It is not entirely clear why this misclassification persisted. One possible explanation could be the biochemical similarities between the two classes; alternatively, this may well be due to the low representation of Langerhans islets in the training data set used to build the model. However, the overall percentage of Langerhans islets between the lobules is relatively low (1.12 ± 0.32 [%][Bibr ref30]) and does not influence model prediction ability on the tissue level.

In summary, our approach demonstrated significant promise, especially considering that we trained the classifier with only 31 pancreatic sections (detailed information provided in the Supporting Information). The overall pixel-level prediction accuracy reached 0.77, which is a commendable result, particularly given that the majority of analyzed classes exhibited true positive rates nearing 80%.

For example, in tissue no. 19, representing a section from a KC mouse, six out of seven classes (except necrosis) can be detected (Figure 4).

Our model first classified PanINs scattered throughout tissue no. 19. Historically, ductal cells were considered the primary source of PanIN lesions. However, further studies suggest that acinar cells can undergo acinar-to-ductal metaplasia (ADM), acquiring progenitor-like characteristics of the early duct cells (Figure 1). If malignantly transformed owing to the presence of the oncogenic *Kras*, these cells serve as the initial site of PanIN formation.^17,18,31^ Indeed, tissue no. 19 exhibited high levels of ADM. Regions with a high abundance of ADM were classified as PanINs, while areas with scattered ADM – as normal acinar tissue (Figure 4C in the third column of zoomed pictures).

Figure 4 shows numerous PanINs, with some cancerous areas in the largest ducts (Figure 4C, second and fourth columns). This finding is particularly interesting, as the analyzed pancreas was sourced from a KC mouse, which is not typically expected to spontaneously develop pancreatic cancer.^19^ Of course, a finite probability exists that this particular animal had developed pancreatic cancer due to the stochastic nature of genetic mutations. However, it is also known that in *Kras* ^G12D^-mutated cells, senescence is induced, which halts further carcinogenesis.^32^ For cancer initiation and progression, additional mutations such as *Trp53* or *p16INK4A* inactivation, are required.^32^ Therefore, it is possible that in our classification we detected senescent cancer cells.

As previously described, our model successfully classified collagen fibers. Fibrosis is a widespread pathological condition that results from prolonged inflammation in organs and soft tissues. Globally, fibrosis is estimated to contribute to nearly 50% of deaths.^33,34^ In the pancreas, fibrosis is a hallmark not only of pancreatic cancer but also of pancreatitis.^34,35^ Although tissue no. 19 primarily exhibits precancerous characteristics, several fibrotic areas were also identified (Figure 4C, first and second columns). Since fibrosis is a consequence of prolonged inflammation, the accurate detection of inflamed regions, as demonstrated by our model (Figure 4C, third column), plays a significant role in both diagnostics and potential preventive strategies. This ability to identify inflammation highlights the broader applicability of our models, which could be further adapted to detect and monitor various pancreatic pathologies.

### Rapid Pathology Detection Model

To create a rapid pathology detection model, we found the smallest possible number of metrics that provided high pixel level accuracy. Information about the importance of metrics for Random Forest classification was derived from the model based on fingerprint region (Figure 5C). We then developed classification models with an increasing number of metrics (listed in Table S3), according to their importance - starting with the most important one. Leave-one-out cross-validation^36^ was applied for confusion matrix calculation, similar to the detailed model described above. Figure 5A shows accuracy values for models created with different numbers of metrics. Six metrics were identified as providing the best model performance while maintaining a low feature count of 110. The model achieved high accuracy values, and in comparison, with models using 4 and 5 metrics, the predicted images (Figure 6) were of very good quality. Figure 5B presents a table of regions used for metrics creation, also shown graphically on the spectra in Figure 5C, with a description of molecular content corresponding to given bands representing vibration of given functional groups. These spectral regions listed define tissue components crucial for distinguishing the three analyzed tissue classes (pathology, fiber, benign). For example, the spectral region 1346–1325 cm^–1^ corresponds to collagen content, with the 1337 cm^–1^ band linked to vibrations of prolines responsible for collagen structuring.^37^ The 1180–1140 cm^–1^ region, with a maximum at 1160 cm-1, corresponds to C–C and C–OH vibrations. Changes in intensity in this band have been associated with glycosylation and carbohydrate accumulation during cancer progression.^38^ The DNA/RNA spectral region (1180–980 cm^–1^) with a maximum at 1080 cm^–1^ form PO^2–^ phosphate group vibrations with contribution from glycogen (1030 cm-1) and other carbohydrates (1155 cm^–1^) also plays an important role in the classification.^39,40^ As reported by Kondepati et al. changes in the DNA/RNA region can be associated with structural alterations of the DNA in pancreatic cancer.^41^ Additionally, glycogen level variations have also been linked to cancer development, as seen in cervical cancer, where lower glycogen content was observed in neoplastic tissue compared to normal tissue.^42^

To calculate the six selected metrics based on the analyzed regions, including Amide I for normalization, 110 frequencies must be measured using the rapid QCL system to achieve the best possible measurement performance. According to the specifications of a commercially available QCL system, measuring the fingerprint region with a QCL microscope would reduce measurement time by approximately 13-fold compared to the optimized Bruker FT-IR measurements performed in this study (a single measurement tile of 64 × 64 μm takes about 13 s), using the same objective magnification and detector size. Moreover, the QCL system allows for measurements with a broader field of view, utilizing a focal plane array (FPA) detector 300 × 300 pixels, and a 3.5× magnification objective. With these parameters, a tissue section of approximately 2 × 2 cm could be measured in just 6 min.

To accelerate calculation time in our rapid pathology detection model, we not only reduced the number of metrics but also limited the model to 25 trees. Additionally, tissues were measured on cost-effective low-e slides in transflection mode, to minimize operational expenses. The potential of this approach for clinical applications was explored in our previous study.^43^

The three-class rapid model presented in Figure 6A enables fast screening of pathologically altered tissues (e.g., no. 4, 6–9, 14–16, 18–20, 30, 31) from normal tissues (e.g., no. 1–3, 10–12). Importantly, in this model, the pathology group includes both PanINs and cancer regions (Figure 6D). Our model achieved True Positive Rate (TPR) values at the pixel level of 84% and 82% for the pathology and healthy classes, respectively. Performance on the tissue level was evaluated based on the ROC curve and AUC value, which is a threshold criterion based on the number of pixels classified as pathology for each sample. Samples can be classified as either healthy or pathological depending on whether they exceeded or fell below this threshold, determined using a control group of samples. After prediction image filtering using a majority filter, the model achieved an AUC value of 0.98, where a value of 1 indicates perfect classification of all tissues. The calculated standard error (SE) suggests potential reproducibility in classifying new samples.

## Conclusions

This study presents two models for detecting preinvasive and invasive pancreatic neoplasias in mouse pancreatic tissue. Our detailed seven-class model distinguishes normal tissue, premalignant dysplasia (PanINs), and cancerous regions. Moreover, we were able to classify blood, infiltrated, fibrotic, and necrotic regions. The ability to classify these regions using IR opens avenues for further exploration of differences between these classes, potentially leading to the discovery of novel biomarkers or therapies, not only for pancreatic cancer but also for other cancers and fibrotic diseases. Furthermore, this model can be used as a tool to study pancreatic cancer development in mouse models. In the future, the seven-class model could be expanded even further to include additional grading of PanINs or pancreatic cancer subtypes.

The three-class “rapid” model was designed to differentiate tissues with pathological regions from normal tissues, achieving a very high AUC value close to 1, highlighting the potential of this approach. This model could accelerate diagnosis by excluding completely healthy tissues from further analysis, even before histological staining.

Our models demonstrate the potential of IR for faster and more automated diagnosis of PDAC patients.

## Materials and Methods

### Selected Chemicals and Consumables

Eosin with phloxine, and hematoxylin, were purchased from Sigma-Aldrich/Merck, USA; KAPA Fast Genotyping Mix was purchased from Roche Diagnostics GmbH, Germany; ExToPCR and Genomic Mini kits were purchased from A&A Biotechnology, Poland; TAE buffer was purchased from Bio-Rad, USA.

### Experimental Mice

The KPC (LSL-Kras^G12D/+^; LSL-Tp53^R172H/+^; Pdx1-Cre) and KC (LSL-Kras^G12D/+^; Pdx1-Cre) breeding pairs (the generous gifts of Dr. Catherine Hogan) were transferred from Cardiff University to the institutional animal facility at Jagiellonian University in 2018 and have been bred there since. These mice were maintained in a controlled environment with a 12-h light/dark cycle, housed individually or in groups of up to five in cages equipped with environmental enrichment (nesting material, wooden gnawing sticks). They had ad libitum access to a standard rodent diet and water. All animal-related procedures adhered to the ARRIVA guidelines.^44^ Some KPC mouse samples were collected as part of another experiment, for which approval no. 113/2020 was obtained from the II Local Ethics Committee for Animal Experimentation in Kraków, Poland.

Pancreatic tissues and tumors were collected from 19 mice up to 14 months of age, including both males and females (former breeders included), with various combinations of Kras^G12D/+^ and Tp53^R172H/+^ mutations. Mice were humanely euthanized via cervical dislocation or CO_2_ overdose. Mouse ID, genotype, and age are available in the table in Figure S1.

### Mouse Genotyping

Mice were genotyped within the first month of life using the standard genotyping protocol, as previously published.^4^ Briefly, DNA was extracted from tail tips using ExToPCR or Genomic Mini kits (A&A Biotechnology, Poland), following the manufacturer’s instructions. PCR reactions were performed using KAPA Fast Genotyping Mix (Roche Diagnostics GmbH, Germany) and specific primers.^45^

### Histology

The normal pancreatic and tumor tissues were fixed in formalin for 48 h at room temperature, then, dehydrated and embedded in paraffin. The tissues were cut into 4 μm sections and stained with hematoxylin and eosin (H&E) using our standard staining protocol.^46,47^ Tile scans of the whole tissue sections were obtained using a Leica DMi8 light microscope (Leica, Germany) and are presented in the Figure S1. Histopathological assessment was conducted based on the tile scans and previously published criteria.^28,29^

### Fourier Transform Infrared Imaging

Fourier Transform Infrared Imaging was performed using a Bruker Vertex 70v spectrometer, coupled with a Hyperion 3000 microscope and a 64 × 64 Focal Plane Array (FPA). Tissue sections were placed on low-e slides and measured in transfection mode over the 3850–900 cm^–1^ range, with 8 cm^–1^ spectral resolution and zero filling factor of 1. The sample and background were measured with 4 and 64 coaveraged scans, respectively. A 15× objective was utilized with a numerical aperture equal to 0.4, giving a projected pixel size of 2.7 μm. The IR image of measured tissues is presented in Figure S4.

### Data Preprocessing

Raw spectra were denoised using the Minimum Noise Fraction (MNF) algorithm, with 20 bands identified as optimal for reconstruction.^48^ Given the absence of a scattering correction for transflection data, there is a slight risk that edge effects could impact classification predictions near tissue borders. However, these edge pixels were excluded from model training and validation. In the next step, 42 spectral regions (listed in Table S1) were selected for local baseline correction, 42 spectral regions (Table S1) were chosen, and the Rubber band Band method was applied locally. For each chosen selected region, three spectral metrics were calculated, spectral band’s: maximum, center of gravity, and integration. Those values were in the next step normalized to corresponding metrics of the Amide I band for size effect removal. The final number of features called further metrics, was equal to 123.

### Random Forest Classification

Pixels were chosen for model creation and validation based on histopathological annotations–regions of interest (ROIs) were annotated on IR images. The model was validated on pixel and mouse levels. On the mouse level, we applied cross-validation to leave one mouse out, it gave us a number of folds equal to the number of mice–19. On the pixel level, pixels from mice assigned to the model test were randomly chosen from ROIs for Random Forest classifier training. All pixels coming from the test set were used for model prediction power assessment–confusion matrix calculation. Two models were created: a detailed model for PanIN classification (based on 50 trees), and a rapid model for rapid pathology detection (based on 25 trees). To establish the importance metrics for the rapid pathology model, a model with 100 trees was first trained (Supporting Information, Figure S7). For the final rapid pathology detection model, the six metrics with the highest importance were selected for model creation (Table S3). The predictive power of the models was evaluated with confusion matrixes, and for the rapid model, additionally, the Receiver Operating Characteristic (ROC) curve and the area under the curve (AUC) value were used. The standard deviation from AUC (SE) was calculated based on the formulas provided in the Supporting Information. The predicted image from the rapid model was filtered with a majority filter with a window size of 5.

## Supplementary Material



## Data Availability

The raw data supporting the findings of this study have been deposited in RODBUK and are openly available at https://uj.rodbuk.pl under the reference number doi.org/10.57903/UJ/LE4EHK.

## Notes

Details regarding AUC calculation; spectral regions used for the metrics; H&E stained tissues presented in Figures 3C and 6A; mean raw spectra and the mean spectra after processing; and the IR image (amide A band) of the measured tissues (PDF)
