# Supplementary material for: Infrared Imaging Combined with Machine Learning for Detection of the (Pre)Invasive Pancreatic Neoplasia
Source: ACS Pharmacol Transl Sci. 2025 Mar 20;8(4):1096–105. doi: 10.1021/acsptsci.4c00689 (PMC11997891; doi:10.1021/acsptsci.4c00689)
Supplement: Supplementary file 1 [file pt4c00689_si_001.pdf]

Supplementary Information:

Infrared imaging combined with machine learning for detection of the (pre)invasive pancreatic neoplasia

Danuta Liberda-Matyja[1,2#], Kinga B. Stopa[1,3#], Daria Krzysztofik[1,3], Pawel E. Ferdek[4]*, Monika A. Jakubowska[3]* and Tomasz P. Wrobel[1*]

[1] Jagiellonian University, Doctoral School of Exact and Natural Sciences, Prof. St. Łojasiewicza 11, 30-348, Kraków, Poland

[2] Solaris National Synchrotron Radiation Centre, Jagiellonian University, Czerwone Maki 98, 30-392
Krakow, Poland.

[3] Jagiellonian University, Malopolska Centre of Biotechnology, Gronostajowa 7A, 30-387 Kraków, Poland

[4] Jagiellonian University, Faculty of Biochemistry, Biophysics and Biotechnology, Department of Cell Biology, Gronostajowa 7, 30-387 Kraków, Poland

# equal contribution

*Corresponding author name and email address:

Tomasz P. Wrobel - tomek.wrobel@uj.edu.pl

Monika A. Jakubowska - monika.jakubowska@uj.edu.pl

Pawel E. Ferdek - pawel.ferdek@uj.edu.pl

The standard error for the AUC value was calculated as follows:

$$SE(AUC) = \sqrt{\frac{AUC(1-AUC)+(n_1-1)(Q_1-AUC^2)+(n_0-1)(Q_2-AUC^2)}{n_0 n_1}} \qquad (1)$$

where n_0 is the number of samples for positive class, n_1 is the number of other samples:

$$Q_1 = \frac{AUC}{2-AUC},\ Q_2 = \frac{2AUC^2}{1+AUC} \quad (2), (3)$$

Table S1. Spectral regions used for metrics (band maximum intensity, center of band gravity, band integration) creation. Region of Amide I (1586-1705 cm^{-1}) was used for normalization

Metric no.	Band range [cm^{-1}]	
1	940	980
2	1015	1040
3	980	1050
4	1040	1070
5	980	1100
6	1050	1100
7	980	1140
8	1050	1140
9	1100	1140
10	1010	1155
11	1160	1180
12	1140	1180
13	1190	1210
14	1180	1210
15	1210	1280
16	1190	1290
17	1180	1290
18	1210	1290
19	1263	1296
20	1295	1320
21	1295	1325
22	1325	1346
23	1340	1360
24	1360	1390
25	1346	1420
26	1360	1425
27	1420	1478
28	1425	1480
29	1478	1586
30	1490	1590
31	1710	1760
32	2825	2860
33	2800	2880
34	2880	2940
35	2940	2980
36	2800	3025
37	3000	3025
38	2995	3095
39	3025	3100
40	2995	3570
41	3100	3600

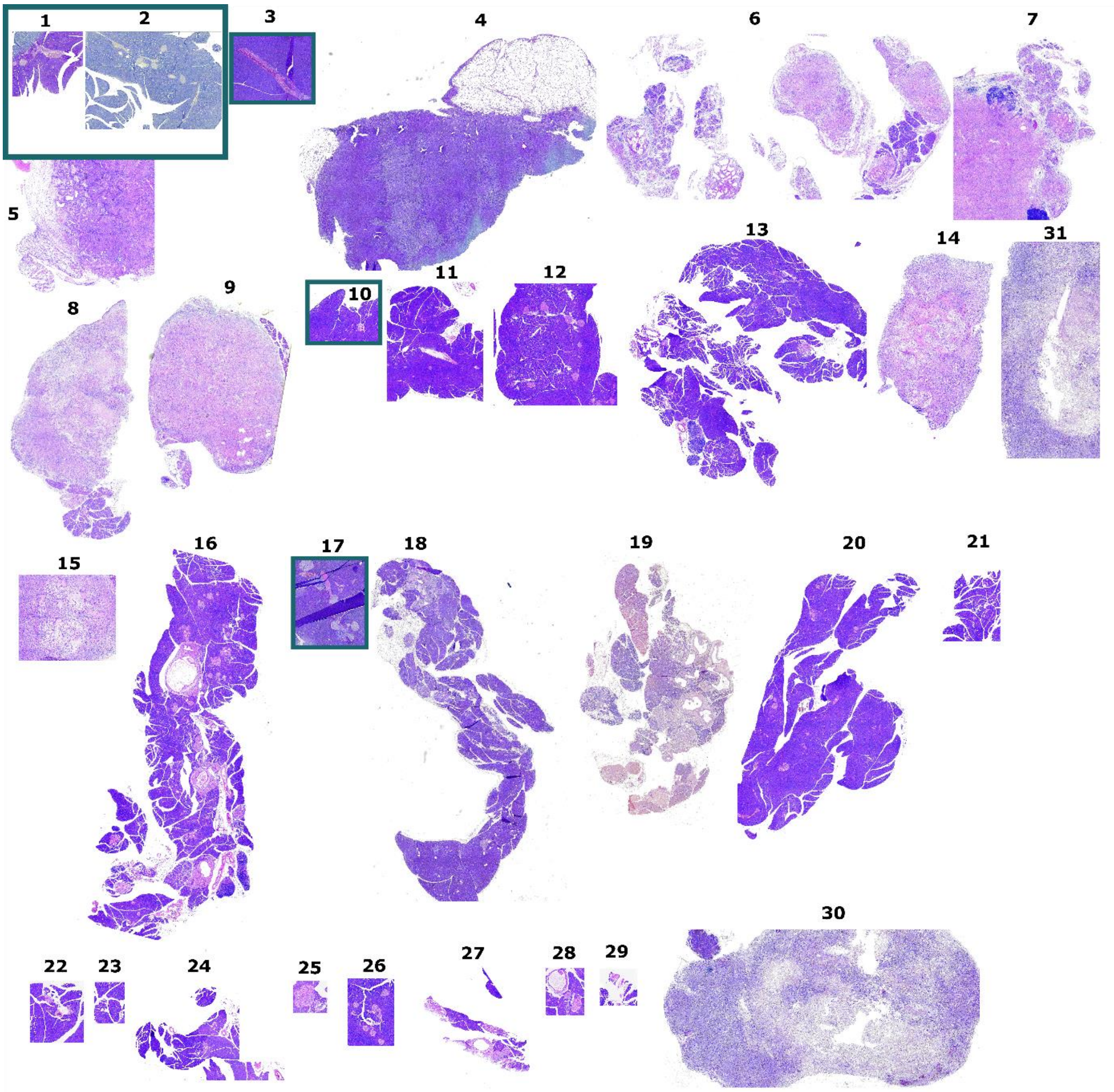

Section number	1, 2	3	4	5, 6, 7	8	9	10	11, 12	13
Mouse ID	XA67	XA90	XA108	XA268	XA272	XA293	XA311	XA329	XA382
Sex	M	F	M	M	M	M	F	M	M
Presence of Kras ^{G12D} mutation	wt	wt	het	het	het	het	het	het	het
Presence of p53 ^{R172H} mutation	het	het	het	het	het	het	het	het	het
Age [months]	5	3	14	4	8	3	6	3	1

Section number	14, 15	16	17	18	19	20	21, 22, 23, 24, 25	26	27, 28, 29	30, 31
Mouse ID	XA405	XB137	XB178	XB181	XB184	XB185	XB298	XB311	XB314	XA8
Sex	F	F	M	F	F	M	F	F	F	M
Presence of Kras ^{G12D} mutation	het	het	wt	het	het	het	het	het	het	het
Presence of p53 ^{R172H} mutation	het	wt	wt	wt	wt	wt	wt	wt	wt	het
Age [months]	2	4	14	13	13	8	9	9	9	

Figure S1. H&E stained tissues. With a green frame, normal tissues were marked. At the bottom of the figure, the description can be found. The total number of mice equals 19, 31 tissue samples were measured in total.

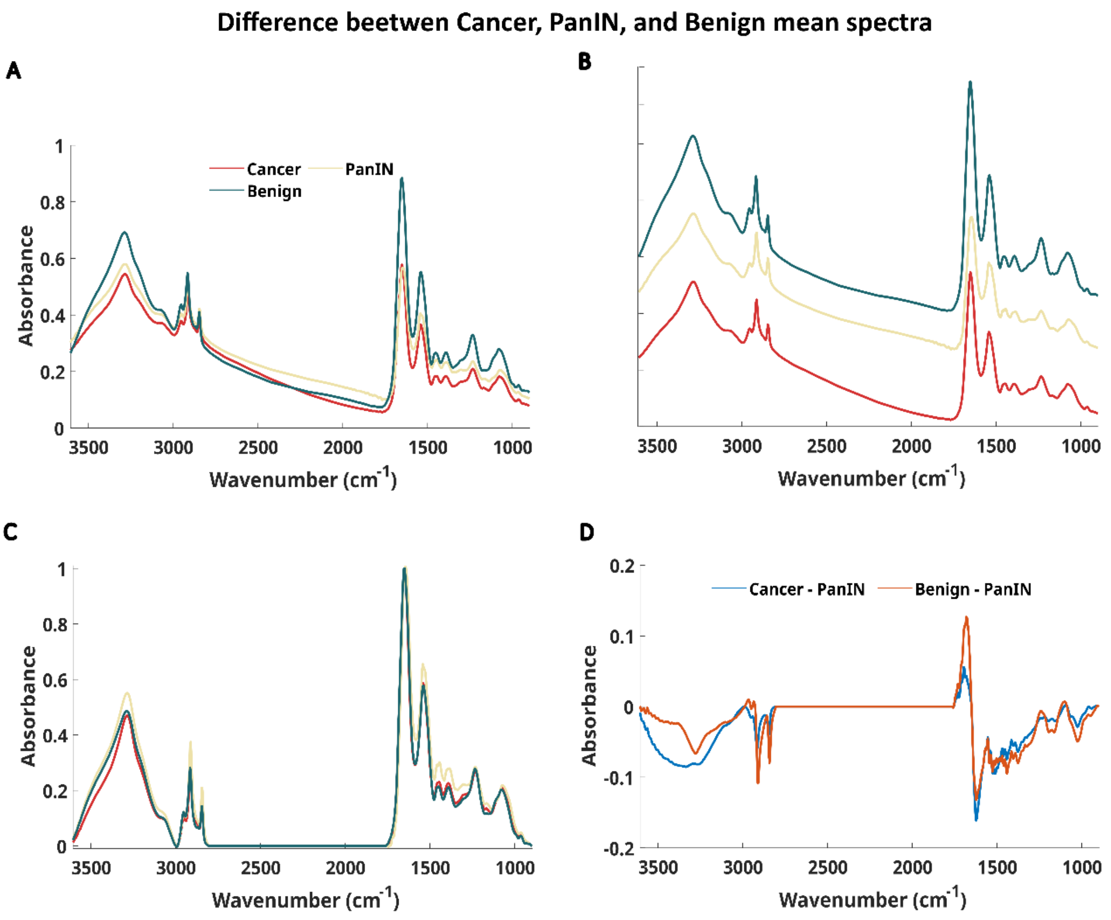

Figure S2. A) raw mean spectra of three classes: Cancer, PanIN, and Benign. B) shifted mean spectra, C) mean spectra after rubber band baseline correction and normalization to Amide I band, D) differences between preprocessed mean spectra.

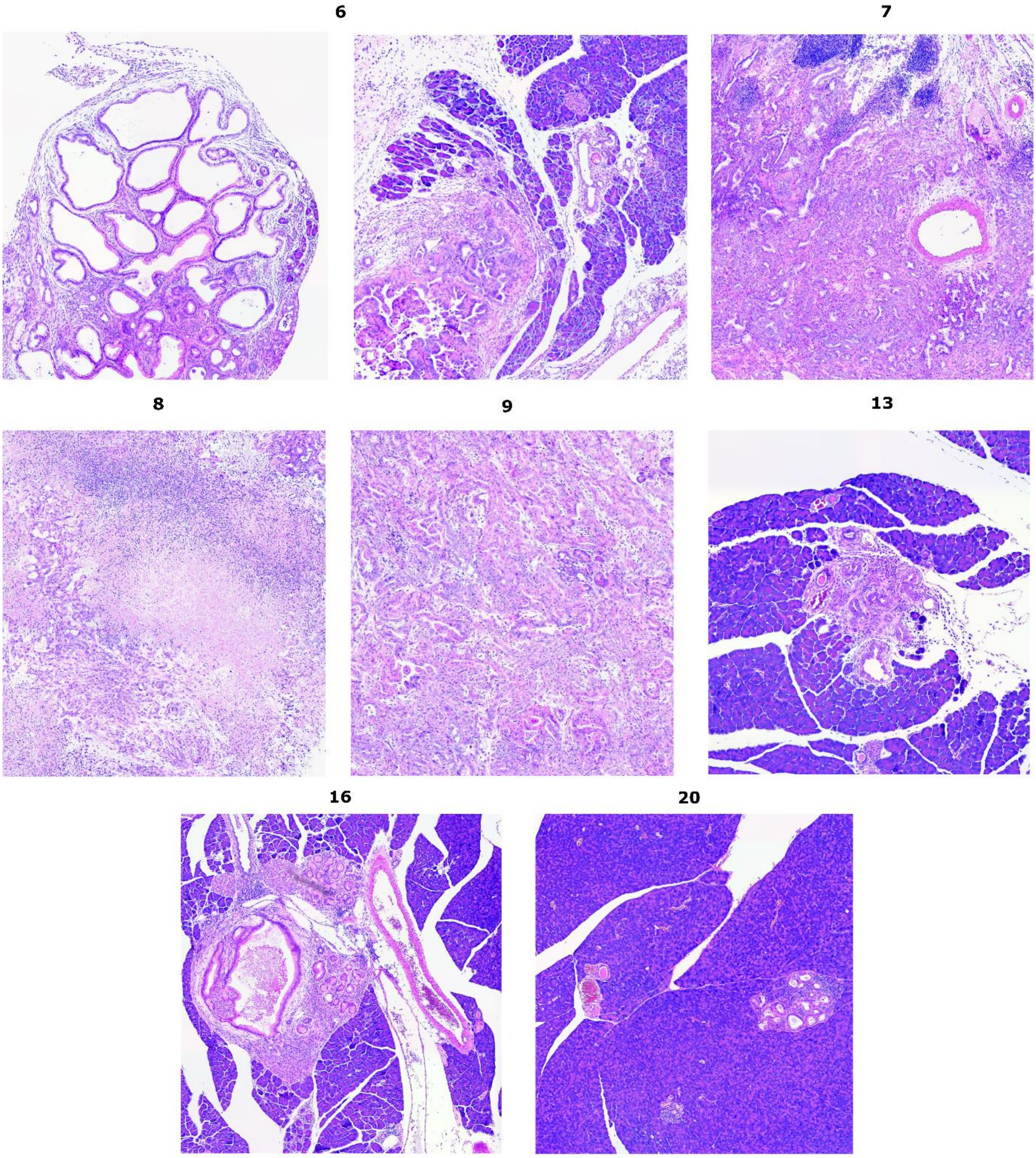

Figure S3. Zoomed H&E stained tissue regions corresponding to zoomed predictions in Figure 3C.

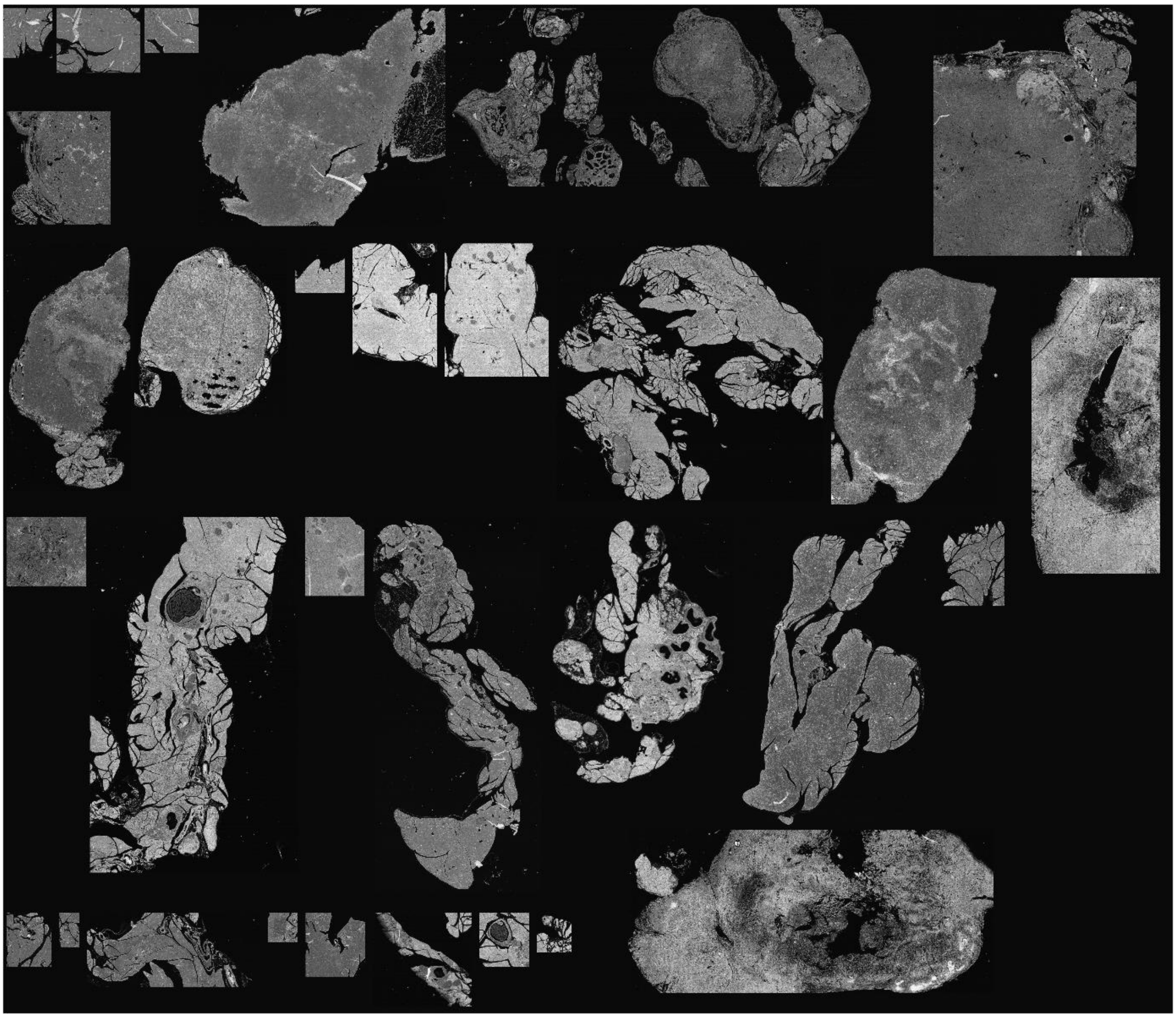

Figure S4. The IR image (Amide A band) of measured tissues.

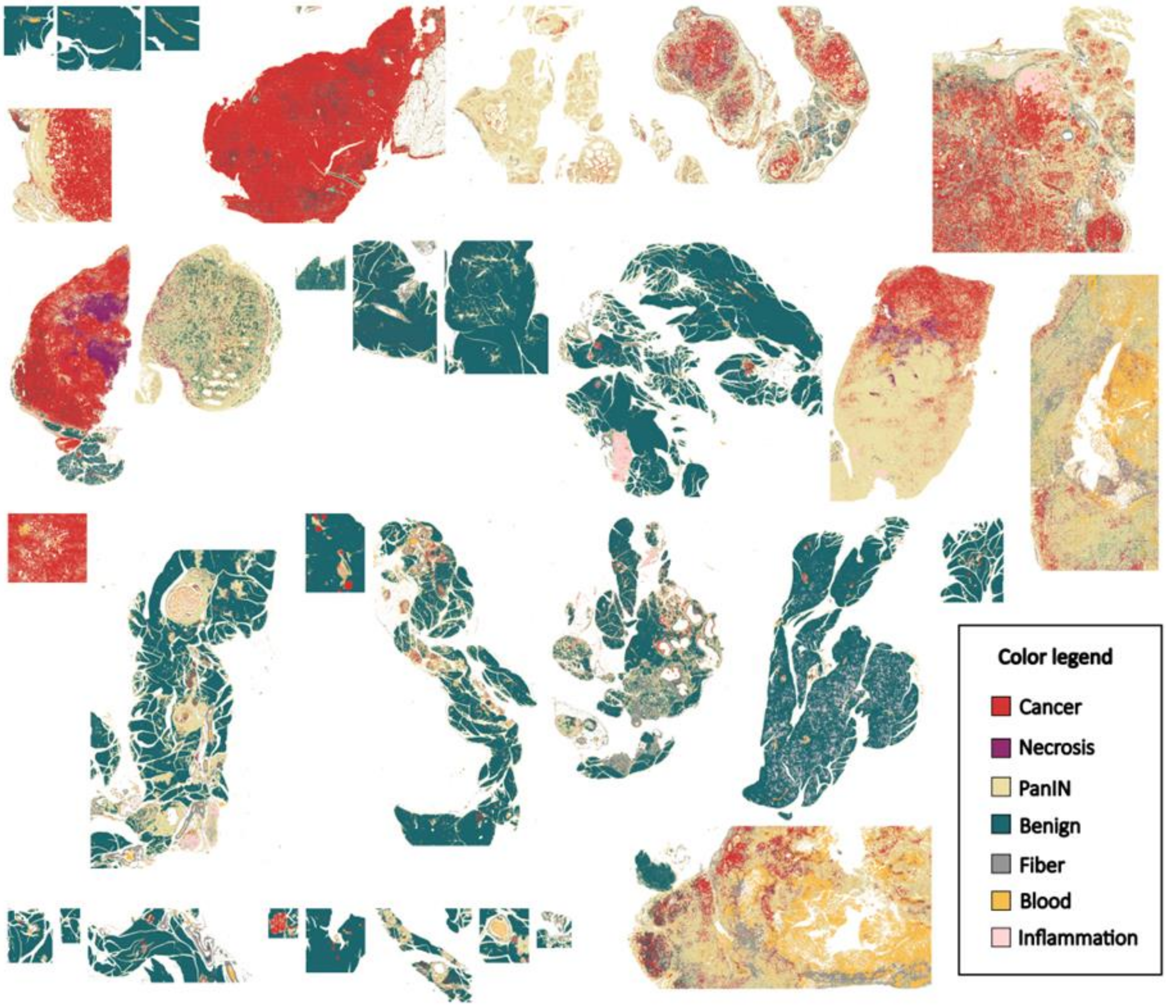

Figure S5. Prediction presented in Figure 2 with whole measured tissue areas visible.

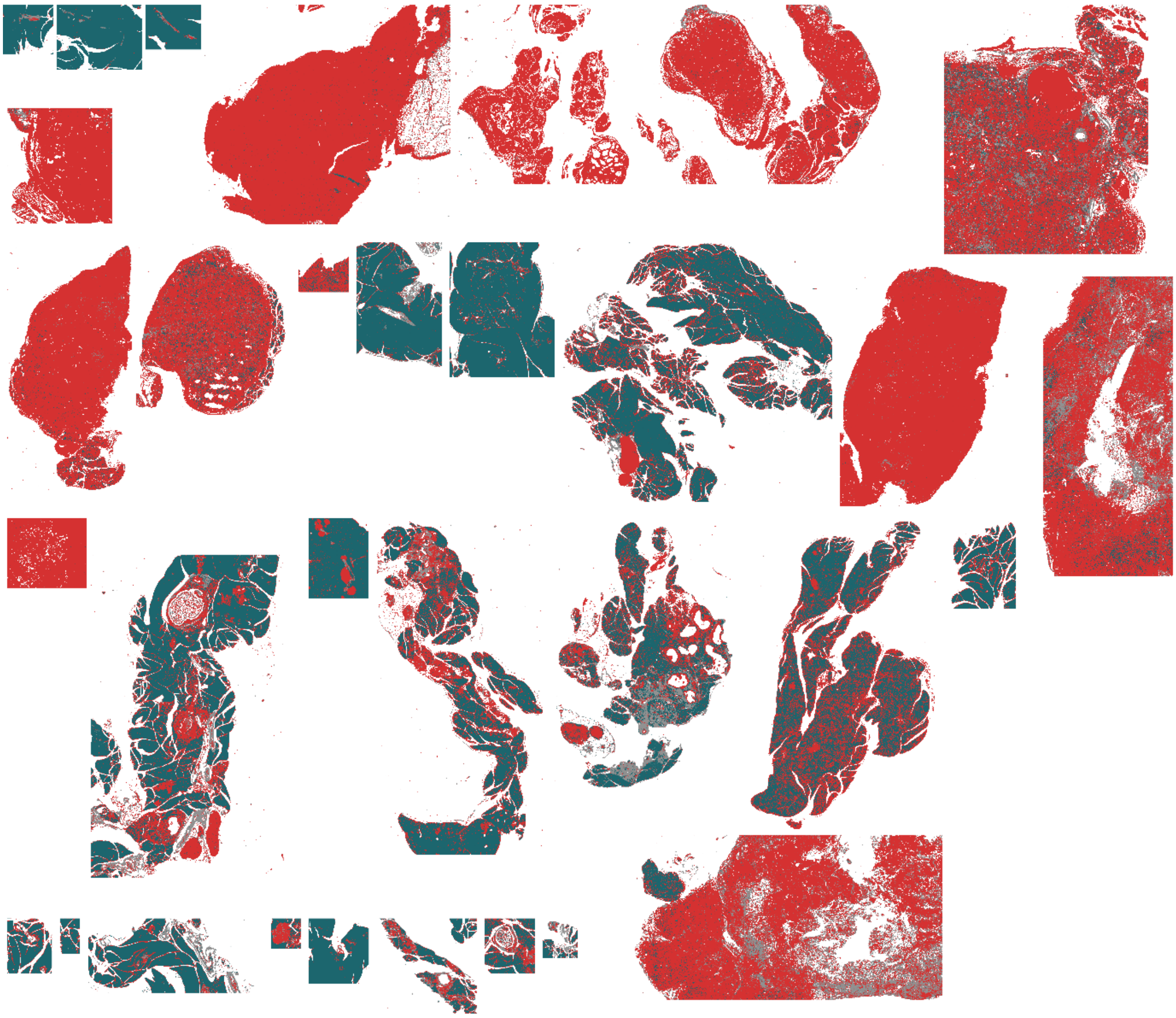

Figure S6. Prediction of mouse tissues with a three class model based on full fingerprint region and 100 trees for optimization of rapid pathology detection model.

Table S2. Spectral regions used for metrics (band maximum intensity, center of band gravity, band integration) creation in rapid pathology detection model. Region of Amide I (1586-1705 cm^{-1}) was used for normalization.

Maximum intensity:

Metric no.	Band range [cm^{-1}]	
1	940	980
2	1015	1040
3	980	1050
4	1040	1070
5	980	1100
6	1050	1100
7	980	1140
8	1050	1140
9	1100	1140
10	1010	1155
11	1160	1180
12	1140	1180
13	1190	1210
14	1180	1210
15	1210	1280
16	1190	1290
17	1180	1290
18	1210	1290
19	1263	1296
20	1295	1320
21	1295	1325
22	1325	1346
23	1340	1360
24	1360	1390
25	1346	1420
26	1360	1425
27	1420	1478
28	1425	1480
29	1478	1586
30	1490	1590
31	1710	1760

Center of band gravity:

Metric no.	Band range [cm^{-1}]	
32	940	980
33	1015	1040
34	980	1050
35	1040	1070
36	980	1100
37	1050	1100
38	980	1140
39	1050	1140
40	1100	1140
41	1010	1155
42	1160	1180
43	1140	1180
44	1190	1210
45	1180	1210
46	1210	1280
47	1190	1290
48	1180	1290
49	1210	1290
50	1263	1296
51	1295	1320
52	1295	1325
53	1325	1346
54	1340	1360
55	1360	1390
56	1346	1420
57	1360	1425
58	1420	1478
59	1425	1480
60	1478	1586
61	1490	1590
62	1710	1760

Band integration:

Metric no.	Band range [cm^{-1}]	
78	1190	1290

63	940	980	79	1180	1290
64	1015	1040	80	1210	1290
65	980	1050	81	1263	1296
66	1040	1070	82	1295	1320
67	980	1100	83	1295	1325
68	1050	1100	84	1325	1346
69	980	1140	85	1340	1360
70	1050	1140	86	1360	1390
71	1100	1140	87	1346	1420
72	1010	1155	88	1360	1425
73	1160	1180	89	1420	1478
74	1140	1180	90	1425	1480
75	1190	1210	91	1478	1586
76	1180	1210	92	1490	1590
77	1210	1280	93	1710	1760

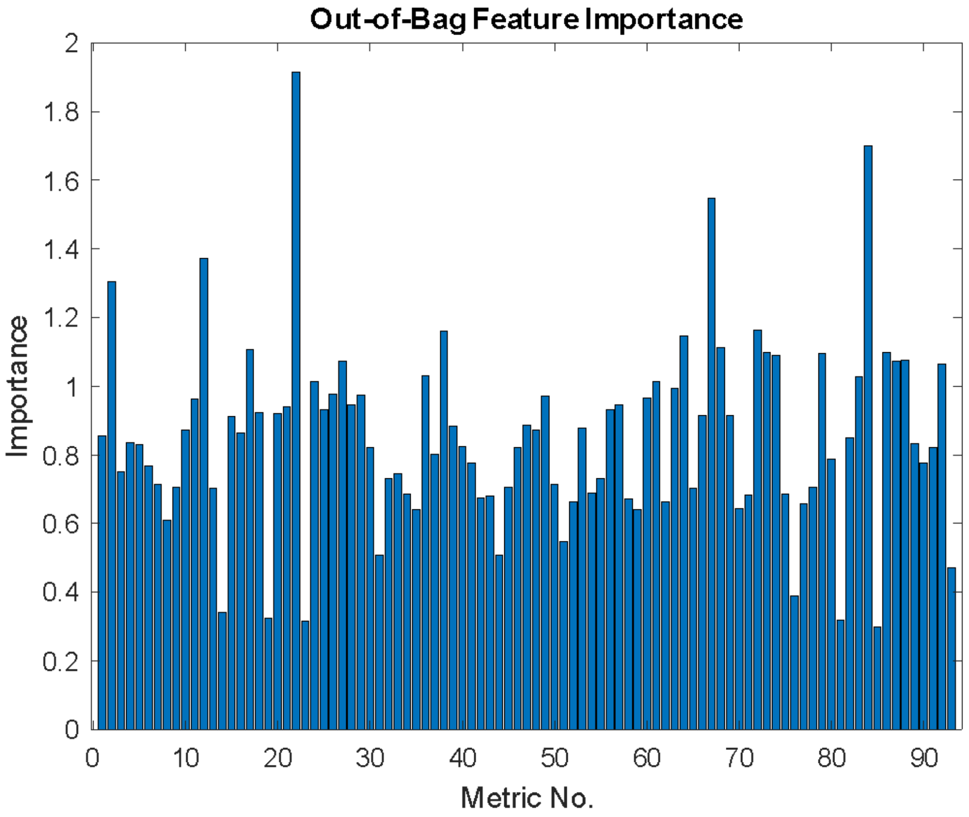

Figure S7. Metrics importance in rapid model creation. The importance of a feature was assessed by out-of-bag (OOB) error, calculated based on feature permutation.

Table S3. Metrics used in rapid model creation sorted according to their decreasing value. Numbers 1 – 31 correspond to the maximum intensity metric, 32–62 correspond to the center of band gravity metric, and 63 – 93 correspond to the band integration metric.

Metrics number used for model creation	Spectral regions no. according to Table S2
1	22
2	22 84
3	22 84 67
4	22 84 67 12
5	22 84 67 12 2
6	22 84 67 12 2 72
7	22 84 67 12 2 72 38
8	22 84 67 12 2 72 38 64
9	22 84 67 12 2 72 38 64 68
10	22 84 67 12 2 72 38 64 68 17
11	22 84 67 12 2 72 38 64 68 17 73
12	22 84 67 12 2 72 38 64 68 17 73 86
13	22 84 67 12 2 72 38 64 68 17 73 86 79
14	22 84 67 12 2 72 38 64 68 17 73 86 79 74
15	22 84 67 12 2 72 38 64 68 17 73 86 79 74 88
16	22 84 67 12 2 72 38 64 68 17 73 86 79 74 88 87
17	22 84 67 12 2 72 38 64 68 17 73 86 79 74 88 87 27
18	22 84 67 12 2 72 38 64 68 17 73 86 79 74 88 87 27 92
19	22 84 67 12 2 72 38 64 68 17 73 86 79 74 88 87 27 92 36
20	22 84 67 12 2 72 38 64 68 17 73 86 79 74 88 87 27 92 36 83
